# Reduced Expression of Uroplakin 1A Is Associated with the Poor Prognosis of Gastric Adenocarcinoma Patients

**DOI:** 10.1371/journal.pone.0093073

**Published:** 2014-04-03

**Authors:** Yan Zheng, Dan-dan Wang, Wei Wang, Ke Pan, Chun-yu Huang, Yuan-fang Li, Qi-Jing Wang, Shu-qiang Yuan, Shan-shan Jiang, Hai-bo Qiu, Yong-ming Chen, Xiao-fei Zhang, Bai-wei Zhao, Cong mai, Jian-chuan Xia, Zhi-wei Zhou

**Affiliations:** 1 State Key Laboratory of Oncology in South China and Department of Experimental Research, Sun Yat-sen University Cancer Center, Guangzhou, People’s Republic of China; 2 Department of Gastric and Pancreatic Surgery, Sun Yat-sen University Cancer Center, Guangzhou, People’s Republic of China; 3 Research Center for Medicinal Biotechnology, Shandong Academy of Medical Sciences, Shandong, P.R. China; 4 Department of Endoscopy, Sun Yat-sen University Cancer Center, Guangzhou, People’s Republic of China; Ospedale Pediatrico Bambino Gesu', Italy

## Abstract

**Background:**

The aim of this study was to investigate the expression and prognostic significance of Uroplakin1A (UPK1A) in gastric adenocarcinoma patients. Functional studies were also analyzed *in vitro*.

**Methodology/Principal Findings:**

Real-time quantitative PCR (RT-qPCR), western blotting, and immunohistochemical (IHC) staining methods were used to analyze the expression of UPK1A in primary gastric adenocarcinoma tissue samples. Compared with matched adjacent non-tumor, the expression of UPK1A in fresh surgical specimens was reduced, which was confirmed by RT-qPCR (*P*<0.01) and western blotting analysis (P<0.01). The paraffin specimens from a consecutive series of 445 gastric adenocarcinoma patients who underwent surgery between 2003 and 2006 were analyzed by IHC staining. The relationship between UPK1A expression, clinicopathological factors, and survival were evaluated. IHC staining analysis revealed that the reduced expression of UPK1A was observed in 224 cases (50.3%). Additionally, the correlation analysis of clinicopathological factors demonstrated that reduced expression of UPK1A was significantly associated with histological grade (P = 0.022), node metastasis (P<0.001) and tumor node metastasis (TNM) stage (P = 0.008) (7^th^ edition of the International Union Against Cancer (UICC)). Furthermore, Kaplan-Meier survival analysis revealed that the reduced expression of UPK1A was significantly associated with poor prognosis (P = 0.043). Cox hazards model analysis indicated that UPK1A expression was an independent risk factor at the 0.1 level (P = 0.094). The function of UPK1A in cell cycle, migration, and invasion was investigated by overexpressing UPK1A in the MKN45 gastric cancer cell line. The elevated expression of UPK1A cells induced G1 phase arrest and significantly inhibited migration and invasion.

**Conclusions/Significance:**

The reduced expression of UPK1A might play a role in the progression of gastric cancer. Thus, UPK1A could be a potential favorable biomarker associated with gastric cancer prognosis.

## Introduction

Gastric carcinoma (GC) is the fourth most common cancer worldwide [1] and the second most frequent cause of cancer-related deaths in China [2], with more new cases in China diagnosed every year than in any other country [3]. Despite improvements in early diagnosis, advanced surgical techniques and combined therapy (surgery, chemotherapy and radiotherapy), distant metastasis and local recurrence cannot be avoided easily in most cases, and the prognosis of gastric cancer remains far from satisfactory [3]. The progression of gastric cancer is considered a multistep process that involves the activation of oncogenes and the inhibition of tumor suppressor genes. Discovering and understanding the molecular and genetic characteristics of these tumor-associated genes would help us to improve the diagnosis and treatment of gastric cancer patients. If the molecular and genetic characteristics of gastric carcinoma could be better understood, the prognoses of patients may be improved and more appropriate therapies may be chosen.

Uroplakins (UPs) play a central role in cellular physiology and cancer [4], [5]. UPs are reportedly diminished and or absent in invasive carcinomas during tumorigenesis [6]. The Uroplakin 1A (UPK1A) gene belongs to the transmembrane 4 super family (TM4SF), also known as the tetraspanin family [7], [8]. The expression of UPK1A is known to be highly specific to normal urothelium [9], and can be observed in the normal genitourinary tract, uterus and prostate [10]. Recently, a study by Kar and colleagues [11] found that UPK1A can be used as a molecular biomarker and is a significant tumor suppressor in esophageal squamous cell carcinoma (ESCC), which demonstrated that UPK1A plays a role in inhibiting cell proliferation, clonogenicity, cell motility, and tumor formation. Furthermore, UPK1A inhibits the nuclear translocation of β-catenin and can serve as an antagonist to matrix metalloproteinase 7 (MMP7), thereby playing a prominent role in tumor suppression [11]. However, the prognostic and clinicopathological significance of UPK1A in human gastric carcinoma (GC) has not yet been investigated.

## Results

### RT-qPCR and Western Blot Analysis

**Figure 1:**
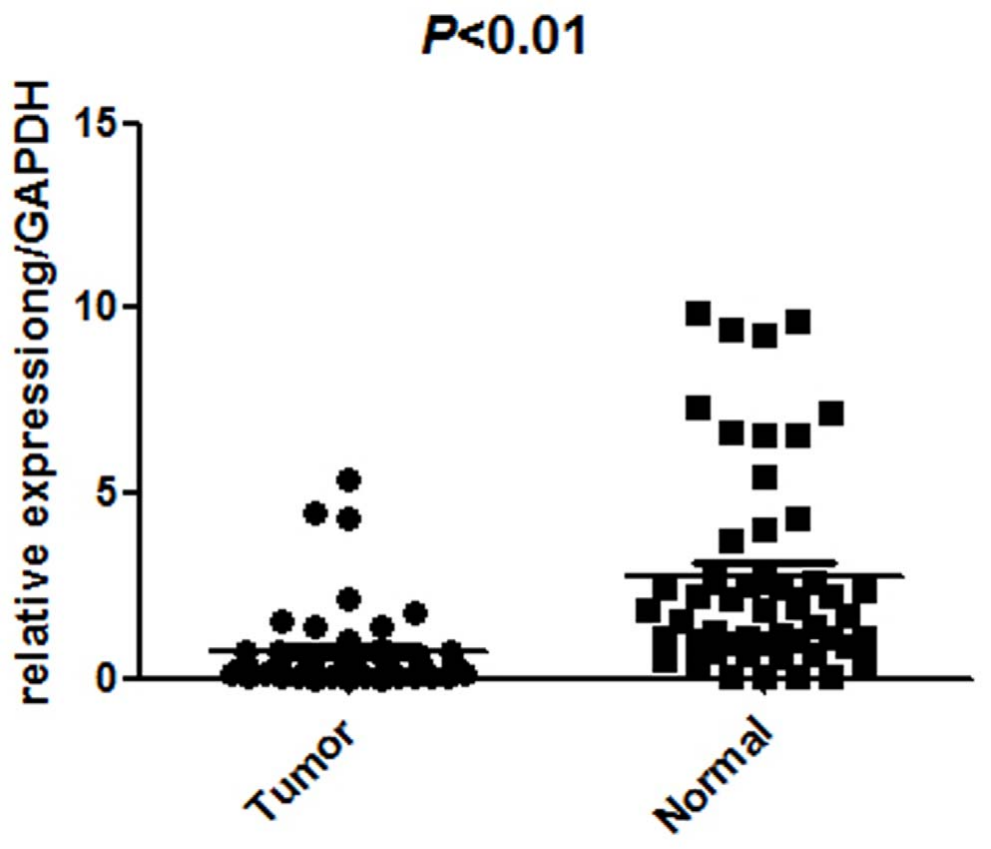
Decreased UPK1A mRNA expression in gastric cancer tumor tissues was detected by RT-qPCR (n = 51, p<0.01, gastric cancer tumor compared with adjacent non-tumor tissue, Wilcoxon matched-pairs signed-rank test). The small box inside the outside box represents the mean.

**Figure 2:**
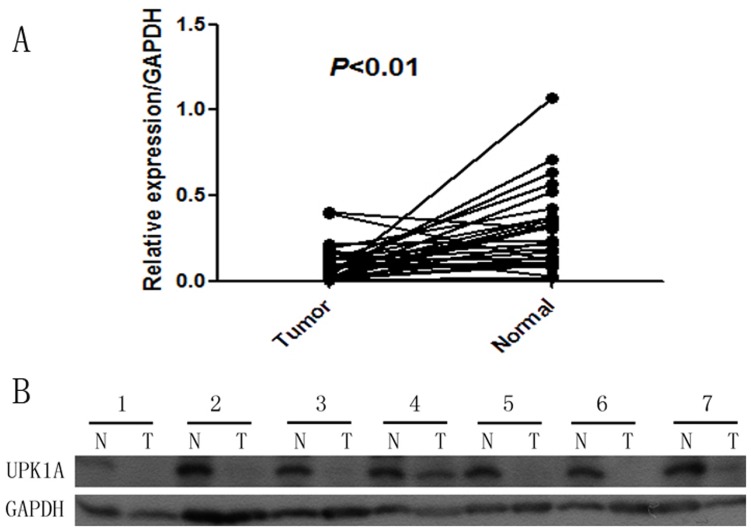
Decreased expression of UPK1A protein in GC tumor tissues was detected by western blotting (n = 29, p<0.01, gastric cancer tumor compared with tissue, Wilcoxon matched-pairs signed-rank test). (A) Expression of UPK1A protein in gastric tumor and adjacent non-tumor tissues. (B) The relative expression of UPK1A in comparison with the expression level of normal paracancerous tissues. (N, normal paracancerous tissues; T, tumor).

Quantitative RT-qPCR was performed on 51 pairs of surgical specimens (tumor and adjacent non-tumor tissue samples) to examine the mRNA expression of UPK1A. There was a significant difference in the average expression level of UPK1A mRNA between the tumor tissues and the paired non-tumors (*P*<0.01, Figure 1), and the expression of UPK1A was lower in tumor tissues. Western blot detection also revealed similar results in protein levels (*p*<0.01, Figure. 2) by testing 29 paired samples, including carcinoma and normal stomach tissue.

### Immunohistochemistry Analysis and Clinicopathological Characteristics

**Figure 3:**
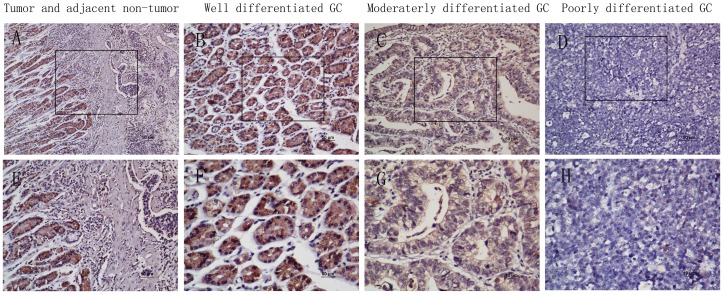
The in situ expression of UPK1A protein in gastric cancer specimens was assessed by immunohistochemistry. (A) and (E), Immuno-staining of a GC tumor and the adjacent non-tumorous area. (B) and (F), Strong UPK1A staining was observed in well-differentiated GC. (C) and (G), Weak UPK1A staining in moderately differentiated gastric cancer. (D) and (H), Negative UPK1A staining in poorly differentiated GC. (A–D with 200× magnification; E–H with 400×magnification).

To further investigate the expression of UPK1A in situ, paraffin-embedded GC tissue blocks (n = 445) were used for immunohistochemical analysis. The results showed that the immuno-staining of UPK1A is varied in tumor tissues and adjacent non-tumor regions. The positive UPK1A expression was localized to the cytoplasm, and reduced cytoplasmic UPK1A was observed in the tumor tissues, especially in the poorly differentially tumor tissues (Figure 3). Two hundred twenty-four cases (50.3%) exhibited low expression of UPK1A (Table 1).

**Table 1:** The Expression of UPK1A and the Clinicopathologic Characteristics of Patients with Gastric Cancer.

Characteristics	number	UPK1A Expression (%)			
		Low	High	χ2	*p* Value
**Gender**				0.499	0.48
Male	301	155	146		
Female	144	69	75		
**Location**				5.483	0.14
Fundus of stomach	181	81	100		
Proximal	67	36	31		
Distant	173	91	82		
Total	24	16	8		
**Histological grade**				7.598	0.022^a^
Well differentiated (G1)	285	151	134		
Moderately differentiated (G2)	73	26	47		
Poorly differentiated (G3)	87	47	40		
**Radical resection**				0.518	0.472
Yes	375	186	189		
No	70	38	32		
**Tumor emboli in vasculature**				1.497	0.221
No	401	198	203		
Yes	44	26	18		
**Tumor invasion (T)**				5.285	0.259
T1	33	11	22		
T2	42	20	22		
T3	82	40	42		
T4a	239	125	114		
T4b	49	28	21		
**Nodal status (N)**				19.104	<0.001^a^
N0	122	43	79		
N1	86	41	45		
N2	103	58	45		
N3	134	82	52		
**Metastasis status (M)**				0.008	0.928
M1	390	196	194		
M2	55	28	27		
**TNM staging 7^th^ ed**				11.96	0.008^a^
Stage I	46	15	31		
Stage II	104	45	59		
Stage III	239	136	103		
Stage IV	56	28	28		

pN, pathological node; pT, pathological tumor; pTNM, pathological tumor/node/metastasis; UPK1A, Uroplakin 1A.

a Statistically significant (*p*<0.05).

**Figure 4:**
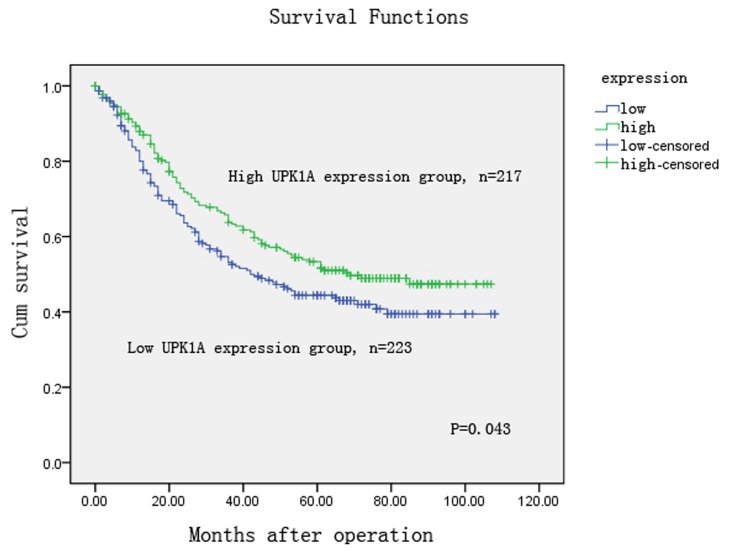
Kaplan-Meier survival analysis of gastric cancer patients (n = 445). The survival rate of patients in the UPK1A-high group was higher than that of the patients in the UPK1A-low group (log-rank test, P = 0.043).

The correlation of UPK1A expression and clinicopathological factors were analyzed according to the IHC detection results of 445 GC samples. We observed that reduced UPK1A expression was significantly associated with histological grade (P = 0.022), lymph node metastases (N3) (P<0.001), and TNM stage (P = 0.008) (Table 1). Furthermore, we investigated the relationship of UPK1A expression and the disease-specific patient survival of the 445 patients with gastric cancer. The median observation period was 54 months (range, 1 to 108months), with 223 surviving patients and 222 cancer-related deaths. The clinical median overall survival (OS) was 54±7.915 (38.487–69.513). The Kaplan-Meier survival analysis indicated that the OS rate was significantly higher in the high UPK1A expression group compared with the low UPK1A expression group (P = 0.043, Figure 4). Univariate analysis revealed that UPK1A expression was a significant prognostic factor for gastric cancer patients (p = 0.045, Table 2). Multivariate Cox proportional hazards model analysis revealed that UPK1A expression was an independent prognostic factors at 0.1 level (P = 0.094, Table 2).

**Table 2:** Univariate and multivariate analyses of overall survival in 445 cases of gastric cancer patients.

Variables	Univariate analysis			Multivariate analysis		
	HR	95% CI	P value	HR	95% CI	P value
**Age**	1.019	1.008–1.031	0.001*	1.014	1.002–1.026	0.024*
**Gender (Male vs. Female)**	1.053	0.795–1.395	0.719			
**Location (Fundus of the stomach/Proximal/Distant/Total)**	0.803	0.700–0.922	0.002*	0.769	0.667–0.887	<0.001*
**Differentiation (G3/G2/G1)**	0.958	0.811–1.132	0.617			
**Radical resection (No vs. Yes)**	4.483	3,312–6.068	<0.001*			
**Tumor emboli in vascular (No vs. Yes)**	3.047	2.116–4.389	<0.001*	1.807	1.241–2.630	0.002*
**TNM staging 7^th^ (I/II/III/IV)**	3.478	2.802–4.318	<0.001*	3.495	2.791–4.375	<0.001*
**UPK1A(high vs. low)**	0.763	0.586–0.994	0.045*	0.796	0.608–1.040	0.095

*Statistically significant (*p*<0.05).

### UPK1A Inhibits Cell Migration and Invasion in the MKN-45 GC Cell Line

**Figure 5:**
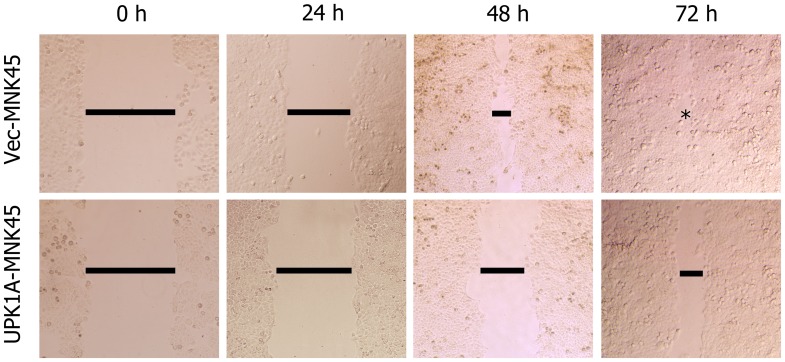
In the wound healing assay, representative images were photographed 0, 24, 48 and 72 hours after scratching. The result demonstrated that the high expression of UPK1A inhibited the cell motility.

**Figure 6:**
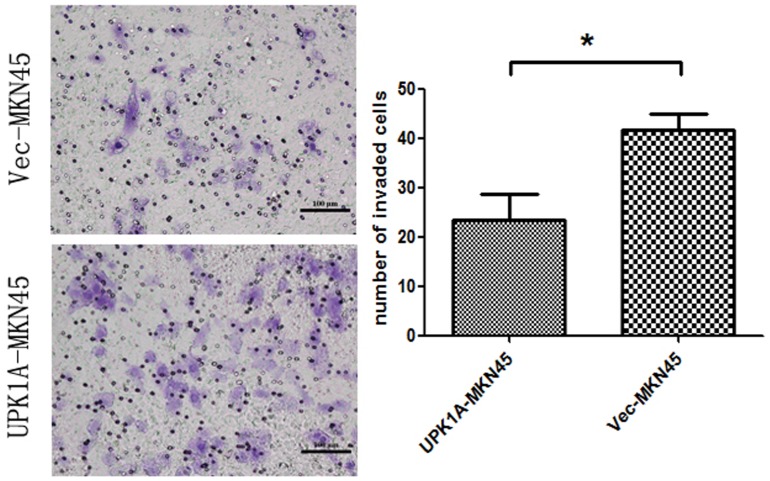
Matrigel invasion assays of vector-MNK45 and UPK1A-overexpressed MNK45 cells. The invading cells were fixed and stained with crystal violet (magnification, 200×) on the left. The right images present the quantification of 10 randomly selected fields. The results represent the mean ± SD of three independent experiments. *, P<0.05.

The wound healing and cell invasion assays were conducted to evaluate the effects of UPK1A expression on cell migration and invasion. In wound healing assays, the migration rate of MKN45-UPK1A cell was significantly reduced compared with MKN45-Vec cells (Figure 5). Consistent with the wound healing assay results, UPK1A also significantly inhibited cell invasion through a Matrigel-coated membrane in the invasion assay (p = 0.0407, Figure 6).

### UPK1A Inhibits the Viability of MKN45 Cell Line

**Figure 7:**
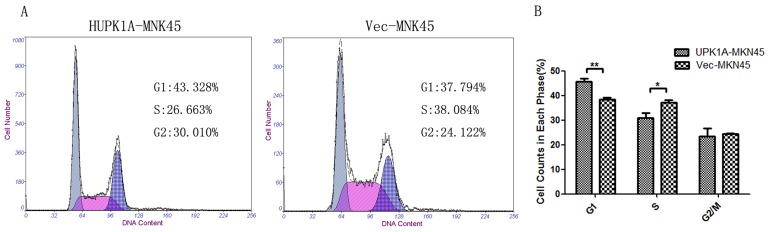
UPK1A overexpression caused G1-S checkpoint arrest in the MKN45 cells. A, The DNA content of Vec-510 and UPK1A-510 cells were compared by flow cytometry. B, The cell proportions from different phases were summarized. The results are expressed as the mean ± SD of three independent experiments. *, P<0.05, **P<0.01.

To understand the effects of UPK1A expression on cell growth, flow cytometry analysis was employed to compare the DNA content of UPK1A-MKN45 and Vec-MKN45 cells. The results indicated that the proportion of G1 phase cells was significantly higher in the UPK1A-overexpressing MKN45 GC cell line (P<0.01), whereas the population of S-phase cells was significantly lower in UPK1A-MKN45 cells (P<0.05). There results suggest that UPK1A induces cell cycle arrest at the G1-S checkpoint (P_G1_ = 0.006, P_S_ = 0.049, Figure 7).

## Discussion

UPK1A belongs to the tetraspanin super family of genes that encode a large number of integral membrane proteins with four transmembrane domains (TMD) [7]. The tetraspanin proteins are quite versatile and are involved in many important cellular functions [7]. The loss of UPK1A expression may result in cell proliferation, metastasis and invasion [11]. Previously, Kar and colleagues [11] uncovered a promising and extensive network of positive and negative regulation of UPK1A, which inhibited the down-regulation of MMP7 to control metastasis, invasion and survival. Based on patient characteristics, etiology, uniform treatment modalities, and long-term follow-up, the present study is the first to systematically evaluate the expression and clinicopathological significance of UPK1A in GC. Our findings indicate that UPK1A may play a role in tumor suppression, and is closely correlated with survival and lymph node metastasis in GC patients. These results are consistent with a number of previous reports [9], [12].

In the present study, the protein and mRNA levels of UPK1A in GC patients were evaluated by western blotting and RT-PCR, respectively. The correlation of UPK1A and clinical outcome was analyzed by using immunohistochemical staining of primary GC tissue. The results revealed that the protein levels of UPK1A were significantly reduced in tumor tissue samples, compared with levels in normal paracancerous tissues (P<0.01). The RT-qPCR findings were consistent with western blotting detections (P<0.01). These results support the previous hypothesis that UPK1A might be a tumor suppressor candidate in gastric cancer. Subsequently, using immunohistochemistry analysis for paraffin specimens, with large series of gastric cancer patients (n = 445), we observed that the low UPK1A expression was associated with histological grade (P = 0.022), lymph node metastases (P<0.001) and UICC stage (P = 0.008). These findings are similar to a previous study [11] that reported an association between low UPK1A expression and lymph node metastasis as well as in esophageal squamous cell carcinoma [11]. Similar findings can be observed in other malignancies [13]. Therefore, UPK1A may serve as a potential tumor-suppressor gene in gastric cancer.

In analyses focused on the survival of patients, the expression level of UPK1A was shown to be a significant predictive variable in surgically resected GC. In the Kaplan–Meier survival analysis, the median OS was significantly longer among patients with high UPK1A expression than among those with low UPK1A expression. Furthermore, in the univariate analysis, UPK1A is a significant factor. However, in the multivariate analysis, UPK1A is an independent risk factor at the 0.1 level. Age, tumor location, tumor emboli in the vasculature, and TNM stage were independent risk factors in the prognosis of gastric cancer patients at 0.05 level. The findings may suggest that decreased UPK1A expression might help identify gastric cancer patients with poor prognosis and more lymph node metastases. However, not all of our findings have been reported elsewhere, and we cannot make a general statement concerning the influence of UPK1A expression on the lymph node metastases of GC.

UPK1A holds β-catenin in the membrane. Therefore, by interacting with kinases, β-catenin is maintained at a low level. If glycogen synthase kinase 3 (GSK3) is inhibited [14], β-catenin will translocate to nucleus and cause carcinogenesis [15]. Kar [11] confirmed the translocation of β-catenin by testing the expression of its downstream targets, including MMP7, c-Jun, c-myc, and n-D1cyclin, all of which were down-regulated. In vitro assays were used to analyze the function of UPK1A. Overexpression of UPK1A inhibited the migration and invasion of MKN45 GC cell line, which was consistent with our clinical data analysis. The down-regulation of UPK1A was significantly associated with lymph node metastasis in GC (P<0.01). Cell cycle analysis revealed that the overexpression of UPK1A led to cell cycle arrest at the G1-S checkpoint. These findings strengthen the hypothesis that UPK1A might play an important role in GC tumor suppression. Furthermore, consistent with our results, a previous study demonstrated that UPK1A could induce cell cycle arrest at the G1-S checkpoint [16]. The mechanism of UPK1A in gastric carcinoma remains to be fully explored, and therefore our future studies will focus on this mechanism and the relationship between UPK1A and MMP7 in GCs.

In conclusion, patients with resected GC and high expression of UPK1A exhibited favorable survival compared with patients with low UPK1A. Our studies demonstrate that the expression level of GC after surgery represents a potential tumor suppressor and valuable prognostic biomarker of GC patients. Further study is needed to fully evaluate whether UPK1A could serve as a potential target for gene therapy in the treatment of gastric cancer.

## Materials and Methods

### Ethics Statement

This study was officially approved by the Ethics Committee of Sun Yat-sen University Cancer Center. Written consent was obtained from patients to participate in study. The patients’ personal data were sought and obtained.

### Patients

Four hundred and fifty-five postoperative patients were retrospectively evaluated between January 2003 and December 2006. The patient selection criteria included the following: (1) gastric adenocarcinoma with histopathological identification; (2) limited or extended surgical history (including gastrectomy+lymphadenetomy); (3) complete and available follow-up data; (4) no chemotherapy and radiotherapy before surgery; (5) no history of other synchronous malignancies or familial malignancy; and (6) no remnant gastric cancer or recurrent gastric cancer. The surgical procedures followed the Japanese Gastric Cancer Association (JGCA) guidelines [17] and were performed by experienced surgeons.

### Tissue Specimens

Between October 2011 and April 2012, at Sun Yat-Sen University Cancer Center, 51 fresh gastric cancer tissue specimens and adjacent non-tumor tissue samples were obtained. The patients exhibited histologically proven GC which was previously untreated with no distant metastasis. The fresh tissue samples were placed into RNA-later (Ambion Inc., USA) once resected from patients and were stored at 4°C overnight to allow thorough penetration of the tissues. Next, the samples were frozen at 80°C until RNA extraction. The pathological examination verified both the tumor tissue and the adjacent non-tumor tissue.

Paraffin-embedded samples were obtained from 445 gastric carcinoma patients who were included as mentioned above. The World Health Organization (WHO) classification criteria were used to differentiate the histological grade of each tumor. The stage was recorded based on the 7th edition of the International Union Against Cancer (UICC) Tumor-Node-Metastasis (TNM) staging system.

### Extraction of Total RNA and RT-qPCR

According to the manufacturer’s protocol, TRIzol solution (Invitrogen, USA) was used to extract total RNA. The DNA contamination was eliminated by using RNAse-free DNAase. Two micrograms of total RNA was used for the reverse transcription (RT) reaction to synthesize first strand cDNA, which was used as a template for RT-qPCR detection. For the evaluation of the relationship between GAPDH (internal control) and UPK1A, the primer sequences were as follows: for UPK1A, 5′-TGCCATCTTCTGCGGCTTCT-3′ (F); 5′-ATCACGGTGGGTGTAGGACG-3′; and for GAPDH, 5′-CTCCTCCTGTTCGACAGTCAGC-3′ (F) and 5′-CCCAATACGACCAAATCCGTT-3′ (R). An Applied Biosystems (ABI 7900HT) RT-qPCR machine that measured the binding of SYBR Green I to double-stranded DNA was used to perform gene-specific amplification. The procedure of amplification was as follows: initial step at 95°C for 10 min, followed by 45 cycles of 95°C for 30 sec and 60°C for 60 sec. The instrument’s software (SDS2.0) was used to calculate the relative quantity of the amplified samples.

### Western Blotting Analysis

The fresh paired tissue samples including tumor and non-tumor tissue were frozen in RIPA lysis buffer. The lysates were cleared by centrifugation (12,000 rpm) at 4°C for 15 min when the samples were used. Protein samples of approximately 30 ug were run on a 12% SDS-PAGE gel. The proteins in the gel were transferred to PVDF membranes. Five percent non-fat milk was used to block non-specific binding sites for 60 min. The membranes were incubated with the primary polyclonal antibody against UPK1A at a 1∶ 800 dilution overnight at 4°C. The membranes were washed with PBST three times every 10 min. The membranes were probed with HRP-conjugated secondary antibody (at a 1∶2000 dilution) for 60 min at room temperature. The membranes were washed with PBST three times, and the bands were developed with an enhanced chemiluminescence system (ECL, Pierce).

### Immunohistochemistry

The formalin-fixed, paraffin-embedded GC surgical tumor samples were sectioned in 2 um slices. The samples were the deparaffinized and rehydrated using graded ethanols. The procedure of antigen retrieval was as follows: The slides were boiled in EDTA (1 mM; pH 8.0) for 15 min in a microwave oven. Endogenous peroxidase activity was blocked with 0.3% hydrogen peroxide solution for 10 min at room temperature. After rinsing with PBS, the slides were incubated overnight at 4°C with a 1∶600 dilution of goat anti-UPK1A polyclonal IgG antibody (Santa Cruz, USA). After three washes in PBS, the samples were incubated with a biotinylated secondary antibody (Zhongshan Golden Bridge Biotech, Beijing, China) for 30 min at room temperature. Finally, 3,3′-diaminobenzidine tetrahydrochloride (DAB) was used to develop the visualization signal and all of the slides were counterstained with hematoxylin.

### Semi-quantitative Methods

Three observers who were blinded to the patients’ clinical outcomes analyzed the specimens (Z.Y., W.DD., and W.W.). Conflicting diagnoses among the observers occurred in less than 10% of the examined slides. After further review, consensus was reached. The immune staining of the total UPK1A was calculated as the sum of the staining intensity and positive percentage (the percentage of the positively stained tumor cells). The scores of staining intensity were as follows: “3” (strongly stained; strikingly positive at low magnification), “2” (moderately stained; visible at low magnification), ‘‘1” (weakly stained; visible at high magnification), or ‘‘0” (no staining). The positive percentage was scored as ‘‘3” (>50%, diffuse), ‘‘2” (25–50%, focal), ‘‘1” (5–25%, sporadic), or ‘‘0” (<5%, negative). The immunostaining scores of the total UPK1A were defined as the value of percent positivity score × staining intensity score. The range of scores is from 0 to 9. The high expression level of UPK1A defined as a total score ≥4, and a total score <4 for low expression. According to this definition, the GC patients were divided into a high UPK1A expression group and a low UPK1A expression group.

### Follow-Up

All of the patients were followed at outpatient clinics. They received clinical and laboratory examinations every 3 months for the first 2 years. Over the next 2 years, the patients were followed every 6 months and then annually for an additional 5 years until patient death or loss to follow-up. We defined the overall survival as the time from the operation to the death or last follow-up. Overall survival was used as a measure of prognosis.

### Statistical Analysis

The UPK1A mRNA and protein levels in the tumor tissue and adjacent normal tissue samples were compared using the Wilcoxon matched-pairs signed-rank test. The relationship between UPK1A and clinicopathological characteristics was compared using the chi-square test. The Kaplan-Meier method was used to analyze the overall survival with the log-rank test. The Cox proportional-hazard analysis was used to explore the effect of clinicopathological variables and UPK1A expression on survival by univariate and multivariate analysis. Covariates with a P-value equal or inferior to 0.1 in univariate analysis were applied in the multivariate model, excluding radical resection because it was associated with TNM stage. Hazard ratios and survival rates were reported with their 95% confidence intervals (CIs). To perform statistical calculations, SPSS 17.0 for Windows (SPSS Inc., Chicago, IL) was used. A two-sided P value of 0.05 was considered to be statistically significant.

### Overexpression of UPK1A in MKN45 GC Cell Lines

**Figure 8:**
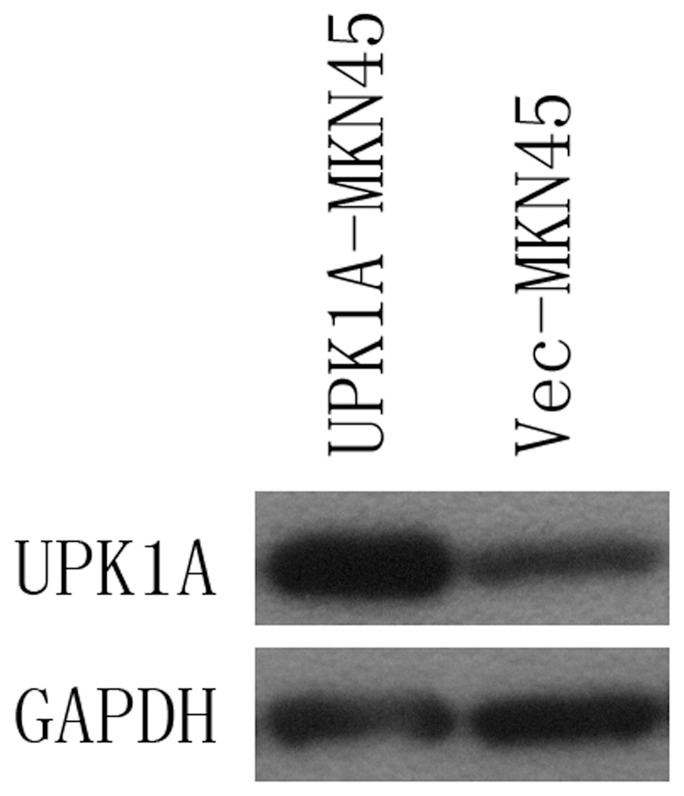
UPK1A expression in the UPK1A-transfected MKN45 cell line (UPK1A-MKN45) was detected by WB. Empty vector-transfected cells (Vec-MKN45) were used as control.

The full-length human *UPK1A* cDNA was cloned into a pcDNA3.1 vector (Invitrogen) and then transfected into the GC cell line MKN45. Empty vector–transfected cells (Vec-MKN45) were used as negative control. Transfections were performed using Lipofectamine 2000 (Invitrogen) according to the manufacturer instructions. Forty-eight hours after transfection, gene expression was confirmed by western blot analysis (Figure 8). Clones with stable UPK1A expression (UPK1A-MKN45) were selected for study. Invasion and migration assays and cell cycle assays were performed.

### Invasion and Migration Assays

A sterile tip was used to wound a cell layer, and the cells were observed after 24, 48 and 72 hours. The photographs were taken by using a microscope at each time point. Meanwhile, according to the manufacturer’s instruction, a Matrigel invasion assay was used. Polycarbonate membrane inserts with 8-mm pores (Corning, USA) were placed in 24-well cell culture plates in a chamber system. A thin layer of 0.5 mg/ml Matrigel Basement Membrane Matrix (BD Biosciences, Bedford, MA) was used to cover the membrane of the inserts. The cells were incubated at 37°C on the Matrigel layer for 36 hours. Subsequently, 75% methanol was used to fix the cells for 30 hours. The cells on the upper surface were removed, and the cells on the lower surface were stained with 0.5% crystal violet containing 20% methanol for 60 minutes. The stained cells were counted in 10 fields under a 20X objective lens. Each experiment was performed in triplicate.

### Cell Cycle Assay

The cell cycle analysis was conducted at 48 hours after the cells were infected. The UPK1A-MKN45 and Vec-MKN45 were washed twice with ice-cold PBS; subsequently the cells were fixed by ice-cold 75% ethyl alcohol at 220°C for one hour. Ice-cold PBS (500ul) was used to resuspend the cells. Subsequently, the cells were incubated with RNase in a 37°C water bath for 30 minutes. Propidium iodide was used to incubate cells in the dark at 4°C for 30–60 minutes. Finally, the cells were analyzed by a flow cytometer (Beckman, USA).
